# Anatomical pancreatic variants in intraductal papillary mucinous neoplasm patients: a cross-sectional study

**DOI:** 10.1186/s12876-022-02465-w

**Published:** 2022-08-21

**Authors:** Katarina Johansson, Harri Mustonen, Hanna Seppänen, Tiina E. Lehtimäki

**Affiliations:** 1 Department of Radiology, HUS Diagnostic Center, University of Helsinki and Helsinki University Hospital, PO Box 340, 00029 HUS Helsinki, Finland; 2 Department of Surgery, University of Helsinki and Helsinki University Hospital, PO Box 440, 00029 HUS Helsinki, Finland; 3 Translational Cancer Medicine Research Program, University of Helsinki, Helsinki, Finland

**Keywords:** Intraductal papillary mucinous neoplasm (IPMN), Meandering main pancreatic duct (MMPD), Ansa pancreatica, Worrisome features, MRI

## Abstract

**Background:**

No previous studies have examined the possible relationship between intraductal papillary mucinous neoplasm (IPMN) and the developmental ductal variations of the pancreas, such as an ansa pancreatica and a meandering main pancreatic duct (MMPD).

**Methods:**

This retrospective cross-sectional study enrolled 214 patients, 108 with IPMN disease and 106 subjects from a community at the tertiary care unit. The main pancreatic duct (MPD) was evaluated in the head of the pancreas by its course, which were non-MMPD: descending, vertical, and sigmoid, or MMPD including loop types, reverse-Z subtypes, and an N-shape, which was identified for the first time in this study. IPMN patients were also evaluated for worrisome features (WF) or high-risk stigmata (HRS), and the extent of IPMN cysts.

**Results:**

Among IPMN patients, 18.4% had MMPD, which we observed in only 3.0% of the control group (*P* < 0.001). Patients with MMPD were more likely to belong to the IPMN group compared with non-MMPD patients [odds ratio (OR) 6.4, 95% confidence interval (CI) 2.2–24.9]. Compared with a descending shape MPD, IPMN patients with an N-shaped MPD were more likely to have a cystic mural nodule (OR 5.9, 95% CI 1.02–36.0). The presence of ansa pancreatica associated with more extent IPMN disease (OR 12.8, 95% CI 2.6–127.7).

**Conclusions:**

IPMN patients exhibited an MMPD more often than control patients. Ansa pancreatica associated with multiple cysts. Furthermore, an N-shape in IPMN patients associated with cystic mural nodules, suggesting that this shape serves as a risk factor for more severe IPMN.

**Supplementary Information:**

The online version contains supplementary material available at 10.1186/s12876-022-02465-w.

## Background

Intraductal papillary mucinous neoplasms (IPMNs) of the pancreas are cystic changes that carry a risk of malignant transformation [1]. IPMN disease falls into three types: branch-duct IPMN (BD-IPMN) in which cysts develop in the branch ducts, main duct IPMN (MD-IPMN) in which the main pancreatic duct (MPD) widens, and mixed-type IPMN (MX-IPMN) in which both cysts develop and the MPD is larger than normal [2]. MX-IPMN and MD-IPMN more often accompany malignant transformation [1, 3, 4]. Additionally, certain worrisome features (WF) and high-risk stigmata (HRS) indicate a higher risk for cancer [3].

During gestation, the ventral and dorsal pancreatic buds first develop separately and then fuse normally with their corresponding ducts [5, 6]. However, there are several developmental variations that can alter the duct configuration. The duct of Santorini, which drains to the minor papilla, is an accessory pancreatic duct that can also be prominent or rudiment [6]. In 30–32% of individuals, the duct of Santorini disconnects from the minor papilla [7, 8]. Furthermore, the configuration of the duct can vary. At the fusion point of the ducts of Santorini and MPD, the MPD may diminish in caliber or develop a loop configuration [6]. Ansa pancreatica is a rare variant, whereby the duct of Santorini takes on a curved or looped course after its fusion point with the MPD [8–10]. Ansa pancreatica can terminate at or near the minor papilla without connecting to the duodenum [8]. Several studies observed an association between ansa pancreatica and pancreatitis [9, 11–13].

Pancreas divisum is the most common congenital anomaly of the pancreatic ductal system, reported in 4–14% of the population [6, 7, 14, 15]. Pancreas divisum results when the ventral and dorsal pancreatic ducts fail to fuse [7]. Four different types of pancreas divisum have been identified: complete [7], incomplete [15], absent ventral duct [16, 17], and reverse pancreas divisum [6, 15, 18–20]. Santorinicele, a focal dilatation of the terminal portion of the duct of Santorini [15], primarily associates with pancreas divisum and a relative obstruction at the minor papilla [7, 21], although it can appear in individuals who do not have pancreas divisum [22, 23].

In addition to the abovementioned developmental variations in ductal anatomy, variations also exist in the course of MPD at the pancreas head. Meandering MPD (MMPD) occurs when the MPD at the head of the pancreas forms two or more tight extrema horizontally observed in coronal images in magnetic resonance cholangiopancreatography (MRCP) sequences, making a loop or so-called reverse-Z type shaped hairpin curves, and not accompanied by other pancreatic ductal anomalies [24]. Gonoi et al. estimated that MMPD associates with pancreatitis [24]. To our knowledge, no previous research examined such variations and their association with IPMN disease.

Here, we aimed to determine if developmental variations and variations in the course of the pancreatic ducts associate with IPMN disease. We also aimed to determine if duct variations associate in any way with the severity of IPMN disease based on the presence of WF and/or HRS and the extent of cysts on the pancreas.

## Methods

### Patient population

**Fig. 1:**
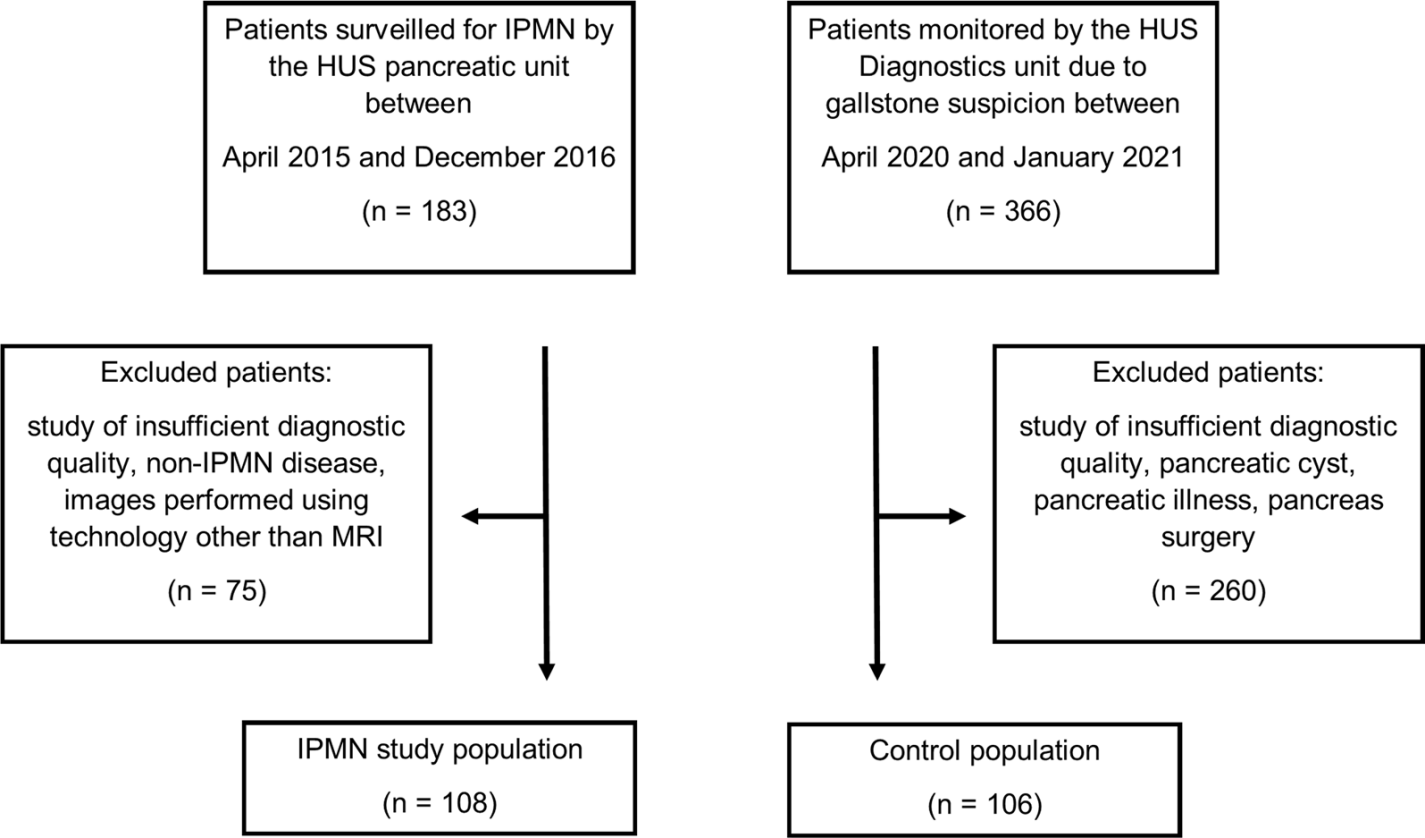
Flowchart of the study population

We used the following inclusion criteria for this study: MRI images from the abdomen that included MRCP sequences, relying on 1.5 Tesla (1.5 T) or 3 Tesla (3 T) images with a normal image quality. For the IPMN group, the inclusion criteria also included a positive working diagnosis of IPMN based on radiological diagnostic criteria (Fig. 1) [3].

Among the control group, we excluded individuals with any pancreatic illness such as a pancreas tumor or cyst, acute or chronic pancreatitis, and patients who underwent pancreas surgery or who presented with diabetes.

Our IPMN study population consisted of 183 patients monitored for pancreatic cysts at the Helsinki University Hospital pancreatic outpatient clinic between April 2015 and December 2016. Our control population consisted of 366 patients scanned based on a suspicion of gallstones at the Helsinki University Hospital between April 2020 and January 2021. Among 183 IPMN study patients, 57 were excluded due to an insufficient diagnostic quality of the imaging (motion artefacts), 5 due to non-IPMN disease, and 13 because the images were based on other modalities. Among 366 control group patients, we excluded 109 because of pancreatic cysts (29.8%), 58 due to motion artefacts, 7 with dilated MPD, 58 with pancreatitis, 8 with pancreatic tumor, 8 who underwent pancreas surgery, and 12 who presented with diabetes (Fig. 1).

This retrospective study was registered with and approved by the Surgical Research Committee of Helsinki University Central Hospital (HUS/333/2019, extended HUS/155/2021) and by the Diagnostic Research Committee of Helsinki University Central Hospital (HUS/211/2020). We were not obligated to secure written informed consent from patients because of the retrospective nature of this study and based on the Act on the Secondary Use of Health and Social data (552/2019).

### MRI imaging techniques

For the IPMN group, MRI examinations were performed using a 1.5 T scanner (Magnetom Avanto Systems, Siemens Healthineers). T2-weighted MRCP three-dimensional (3D) images were acquired through sampling perfection with application-optimized contrast using different flip angle evolution (SPACE). Imaging relied on a repetition time (TR) of 2500 ms and an echo time (TE) of 700 ms, as well as a slice thickness (ST) of 1 mm with a trigged breathing technique.

For the control group, MRI examinations were performed using a 1.5 T scanner (Magnetom Avanto Systems) or a 3 T scanner (Magnetom Skyra Systems, Siemens Healthineers). For the 1.5 T scanner, T2-weighted MRCP 3D images were acquired through SPACE (TR/TE = 4000–4700/705 ms, ST 1.3 mm) with a trigged breathing technique. On the 3 T scanner, T2-weighted MRCP 3D images were acquired through SPACE (TR/TE = 3000–4600/700 ms, ST 1.3 mm) with a trigged breathing technique.

### Image interpretation

Imaging data were retrospectively analyzed independently by two radiologists experienced in pancreatic imaging (KJ and TL, with 5 and 12 years’ experience, respectively). The radiologists were blinded to all clinical information.

**Fig. 2:**
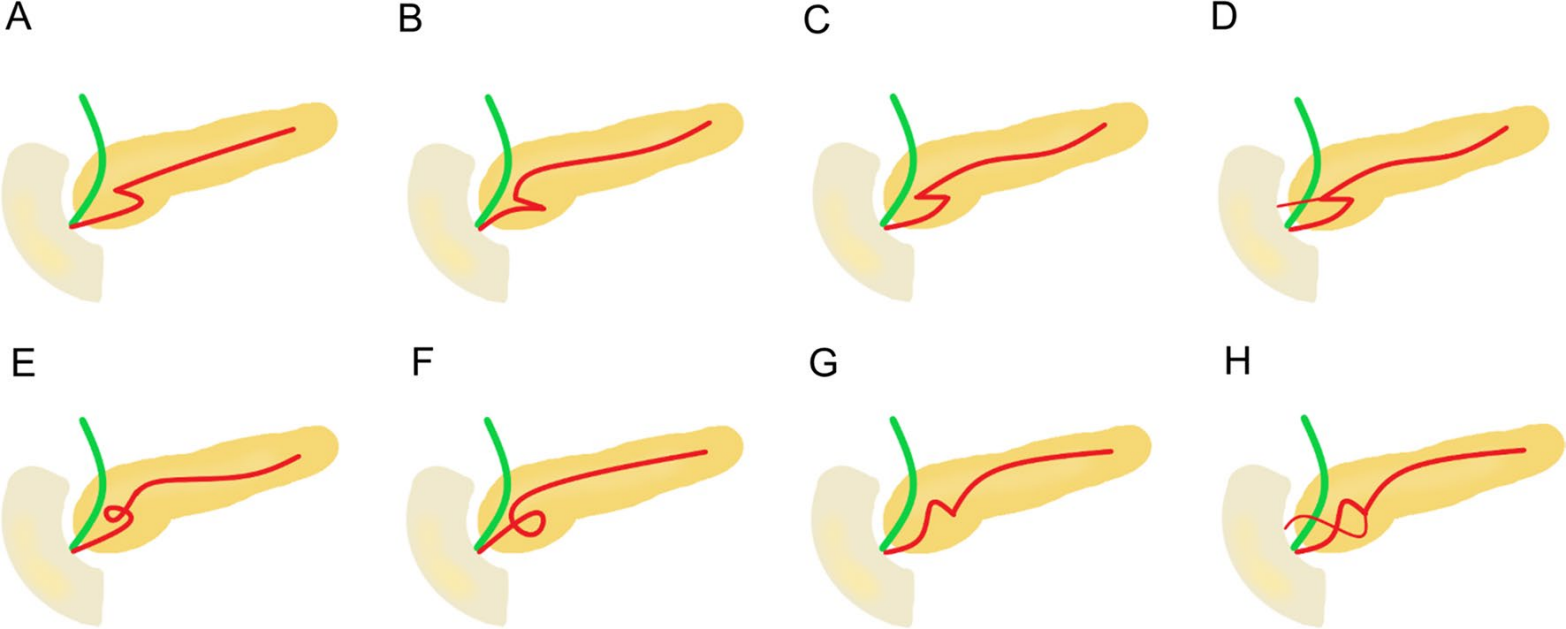
Meandering main pancreatic duct (MMPD) at the head of the pancreas. **a** Reverse-Z subtype C1 where the more upstream turn is tight and < 90° and the second turn is smoother and > 90°. **b** Reverse-Z subtype C2 where the more upstream turn is > 90° and the second turn is tight and < 90°. **c** Reverse-Z subtype C3 where there are two tight and < 90° turns in the horizontal direction along the same plane. **d** C3 subtype with the duct of Santorini which attaches to the right tight turn and drains into the minor papilla. **e** Loop-up configuration. **f** Loop-down configuration. **g** N-shape where the duct forms a deep notch. **h** N-shape where the ansa pancreatica attaches to the notch and drains into the minor papilla

**Fig. 3:**
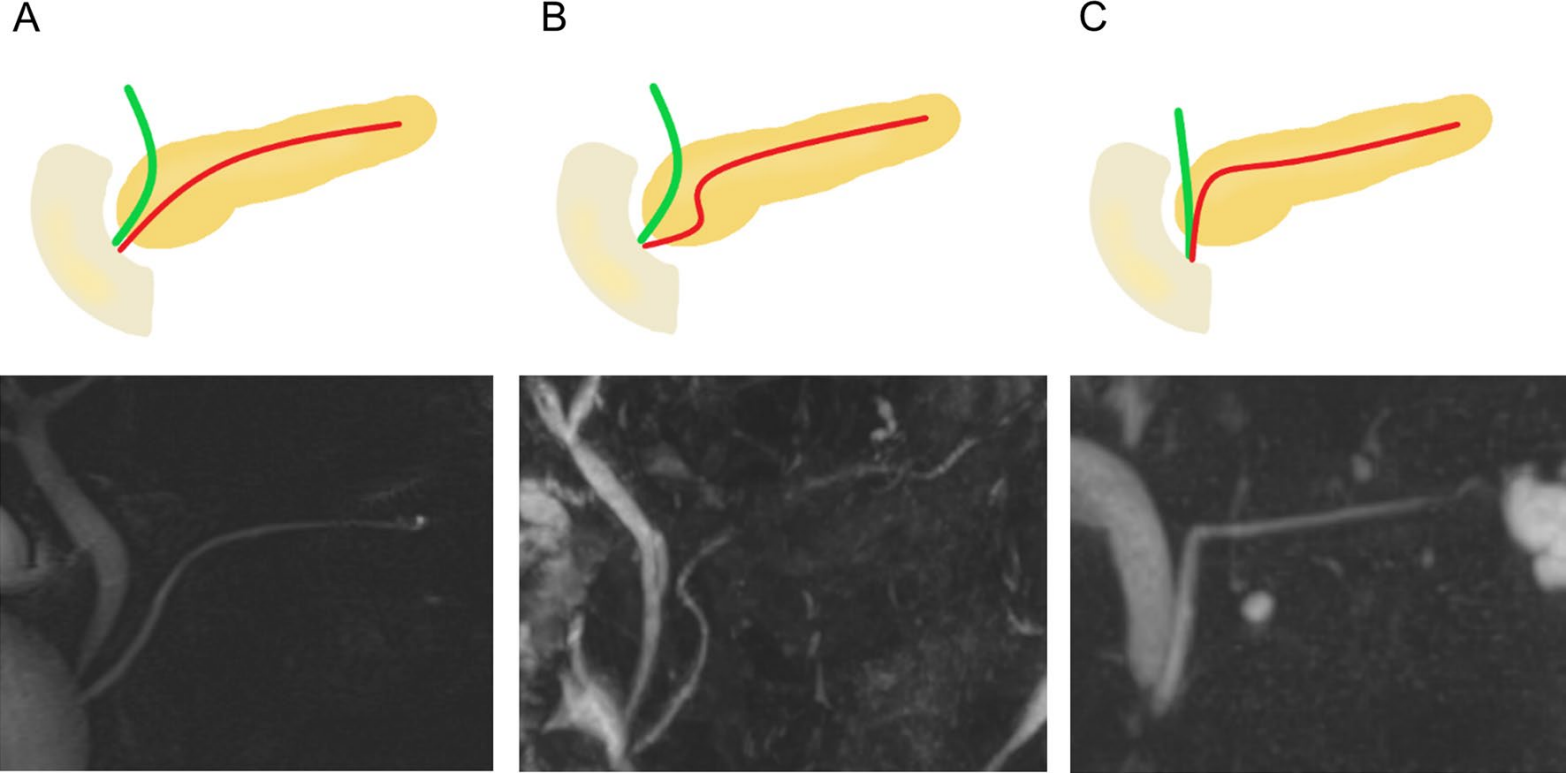
Schematic and MRI images of non-MMPD type courses at the head of the pancreas. **a** Descending course. **b** Sigmoid course with a sigmoid curve. **c** Vertical course draining straight down

In both groups, we determined the presence or absence of the duct of Santorini or the ansa pancreatica (Fig. 2). In the existing duct of Santorini or ansa pancreatica, we determined whether or not the duct connected to the small bowel via minor papilla. We also examined images for the possible presence of the pancreas divisum, categorizing variations as complete, incomplete, reverse, and absent of the ventral duct. The presence or absence of the santorinicele was determined and measured. Possible MMPD at the head of the pancreas was categorized as C1, C2, or C3 reverse-Z types or a loop configuration (Fig. 2) [24]. Reverse-Z subtypes have two extrema in the horizontal direction along the same plane where at least one turn is < 90° without any external compression from other causes. During the study, we found that some patients had a unique MMPD shape at the head of the pancreas, referred to as an N-shape (Fig. 2). In the N-shape, the MPD forms a deep or smaller notch at the head of the pancreas, and the duct of Santorini or ansa pancreatica can unite within that notch. Patients without the aforementioned MMPD or pancreas divisum were categorized based on the course of MPD at the head of the pancreas as descending, vertical, or sigmoid (Fig. 3) [25]. In addition, the caliber of the MPD was measured.

In the IPMN group, we also determined the largest diameter of the cyst, and the location of the solitary or multifocal cysts (uncinate process, head, body, and tail). We also identified cystic and MPD mural nodules (a solid nodule arising from the wall of the cyst or MPD), thickened cyst walls, any abrupt change in the MPD caliber, and the presence of distal parenchymal atrophy, and lymph node enlargement.

### Statistical analysis

We compared anatomical duct variations between the IPMN patient and control groups. We also examined the variation between different types of IPMN disease and meandering ducts among IPMN patients. WF and/or HRS analyses included mural nodules in cysts or in the MPD, an MPD size ≥ 5 mm, an abrupt change in the MPD caliber with distal pancreatic atrophy, and a cyst ≥ 3 cm. In cases with mural nodules also the follow-ups were read to detect which nodules actually were mucin plugs (moving mucin was seen between different scans or the mucin plug decreased in size), and which ones were more likely mural nodules. Also, the possible enhancement was evaluated. The extent of cysts in the pancreas was assessed by comparing equal or more than three-quarters of the pancreas to less than one-half of the pancreas. The cyst locations were divided into four regions: uncinate process, head, body, and tail. Three and four regions of the pancreas meaning equal or more than three-quarters of the pancreas.

We did not analyze the course of the MPD at the head of the pancreas as well as the presence or absence of the duct of Santorini and ansa pancreatica in patients with pancreas divisum. Santoriniceles were evaluated from patients with a pancreas divisum, or a duct of Santorini or ansa pancreatica that ended in the duodenum.

For all analyses, we considered *P* < 0.05 statistically significant using a two-tailed test. Results are reported as the number of patients (proportion of patients), using the mean [and standard deviation (SD)] or median [with either the interquartile range (IQR) or range]. Possible deviations from a normal distribution in continuous variables were tested using the Shapiro–Wilk’s test. All continuous variables had statistically significant deviations from a normal distribution. The Mann–Whitney test was used to assess differences between groups for continuous variables, and the Fisher’s exact test or Fisher–Freeman–Halton test was used for categorical variables. Logistic regression was used to calculate the odds ratios (ORs), 95% confidence intervals (CIs), and *P* values to establish associations between the outcome variable and independent variables. Where indicated in the text, we also used the Firth’s penalized logistic regression. We selected the descending course for the reference group to calculate ORs because it represents the most natural and most common course [25].

All statistical analyses were carried out using either SPSS version 27.0 (IBM SPSS Statistics, version 27.0 for Windows, SPSS, Inc., Chicago, IL, USA) or R version 4.0.3 (Foundation for Statistical Computing, Vienna, Austria).

## Results

The final study population consisted of 214 patients, among whom 108 were in the IPMN group and 106 patients were controls. Table 1 summarizes the patient demographic characteristics for both groups. In the IPMN group, 18.4% had an MMPD, including 4 loop-type cases (2 with a loop up and 2 with a loop down), 8 reverse-Z type cases (7 with C1 subtypes and 1 with a C3 subtype), and 6 N-shape type cases. In the control group, 3.0% had an MMPD, including 1 reverse-Z type (C1 subtype) and 2 N-shape type cases. This difference between groups was statistically significant (*P* < 0.001). Patients with an MMPD in the IPMN group ranged from 49 to 81 years old (mean age 68.3 years, standard deviation (SD) ± 8.2). In the control group, patients with an MMPD were aged 42, 56, and 94 years, respectively. In the IPMN group, 9.3% had pancreas divisum, which fell to 5.7% in the control group. Santoriniceles were observed only in the IPMN group, within which 5 occurred in pancreas divisum patients and one patient had a normal descending duct configuration. The difference between the IPMN and control groups, however, was not statistically significant (*P* = 0.330 for santoriniceles). The IPMN group was older than the control group (*P* < 0.001) and IPMN patients did have a wider MPD (*P* < 0.001).

**Table 1:** Patient demographic characteristics

Characteristics	IPMN group (n = 108)	Control group (n = 106)	*P* value
Mean age at presentation (SD, range)	69 (8, 46–83)	50 (17, 23–94)	** < 0.001**
Sex: female, n (%)	68 (62.9)	62 (58.5)	0.576
MPD width, mean (SD), mm	3.24 (1.65)	2.02 (0.93)	** < 0.001**
Size of the major cyst, mean (SD), mm	19.73 (1.02)	–	
BD-IPMN, n (%)	96 (88.9)	–	
MD-IPMN, n (%)	1 (0.9)	–	
Mixed-type IPMN, n (%)	11 (10.2)	–	
*Developmental variations*			
Pancreas divisum, n (%)	10 (9.3)	6 (5.7)	0.437^1^
Duct of Santorini, n (%)	55 (50.9)	30 (28.3)	
Ansa pancreatica, n (%)	15 (13.9)	10 (9.4)	
Absent duct of Santorini / ansa pancreatica, n (%)	28 (25.9)	60 (56.6)	
*Course of MPD at the pancreatic head*^*2*^	(n = 98)	(n = 100)	
Non-MMPD, n (%)	80 (81.6)	97 (97.0)	
Descending course, n (%)	37 (37.8)	51 (51.0)	
Vertical course, n (%)	10 (10.2)	10 (10.0)	
Sigmoid course, n (%)	33 (33.7)	36 (36.0)	
MMPD, n (%)	18 (18.4)	3 (3.0)	** < 0.001**^3^
Reverse-Z type, n (%)	8 (8.2)	1 (1.0)	
N-shape, n (%)	6 (6.1)	2 (2.0)	
Loop up or down, n (%)	4 (4.1)	0 (0)	

*P* values calculated using the Fisher’s exact test or the Mann–Whitney test. *P* < 0.05 considered statistically significant

BD-IPMN, branch-duct intraductal papillary mucinous neoplasia; IPMN, intraductal papillary mucinous neoplasia; MD-IPMN, main-duct intraductal papillary mucinous neoplasia; MMPD, meandering main pancreatic duct; MPD, main pancreatic duct; SD, standard deviation

^1^Compared to non-pancreas divisum patients

^2^Pancreas divisum patients excluded

^3^Compared to non-MMPD patients

When comparing all MMPD patients to non-MMPD patients, we found an OR of 6.4 of experiencing an IPMN when the patient had an MMPD (*P* < 0.001; Table 2). We also calculated the OR for the association between IPMN and the course of MPD at the head of the pancreas compared to the descending course. When comparing the descending course, the loop configuration (OR 12.4, 95% CI 1.26–1656.69, *P* = 0.028) and the reverse-Z type configuration (OR 7.8, 95% CI 1.65–75.34, *P* = 0.008) had a higher likelihood of experiencing an IPMN. The vertical course (OR 1.37, 95% CI 0.53–3.60, *P* = 0.515), the sigmoid course (OR 1.26, 95% CI 0.67–2.37, *P* = 0.471), and an N-shape (OR 3.57, 95% CI 0.86–20.30, *P* = 0.082) were not statistically significantly associated. When compared to all non-MMPD patients, the loop configuration (OR 10.9, 95% CI 1.1–1452.6, *P* = 0.036) and the reverse-Z type configuration (OR 6.9, 95% CI 1.5–65.4, *P* = 0.011) also had a higher likelihood of experiencing an IPMN. Gender did not appear to affect these results (data not shown).

**Table 2:** The course of the main pancreatic duct (MPD) at the head of the pancreas

Shape	IPMN group (n = 98)	Control group (n = 100)	OR (95% CI)	*P* value
Non-MMPD (%)	80 (81.6)	97 (97.0)	1	
MMPD (%)	18 (18.4)	3 (3.0)	6.40 (2.19–24.94)	** < 0.001**

The Firth’s penalized logistic regression results are shown as odds ratio (OR, outcome is IPMN, control as the reference group) with 95% confidence intervals (CIs) and *P*-value. Comparison between MMPD and non-MMPD patients. Pancreas divisum patients are excluded

CI, confidence interval; IPMN, intraductal papillary mucinous neoplasm; MMPD, meandering main pancreatic duct; OR, odds ratio

Table 3 provides the ORs for the associations between IPMN and patients with the duct of Santorini (OR 3.9, *P* < 0.001) or ansa pancreatica (OR 3.2, *P* = 0.013) compared with the absence of these ducts. When the duct of Santorini connected to the small bowel, the individual was more likely an IPMN patient (OR 5.7, 95% CI 2.1–15.1, *P* < 0.001; Additional file 1).

**Table 3:** Association between the presence or absence of the duct of Santorini or ansa pancreatica and IPMN disease

	IPMN group (n = 98)	Control group (n = 100)	OR (95% CI)	*P* value
Duct of Santorini	55 (64.7%)	30 (35.3%)	3.93 (2.09–7.39)	** < 0.001**
Ansa pancreatica	15 (60.0%)	10 (40.0%)	3.21 (1.28–8.04)	**0.013**
Both absent	28 (31.8%)	60 (68.2%)	1	

Logistic regression results are reported as odds ratio (OR, outcome is IPMN, control as the reference group) with 95% confidence intervals (CI) and *P*-value. The “both absent” group is used as the reference. Patients with pancreas divisum excluded

CI, confidence interval; IPMN, intraductal papillary mucinous neoplasm; OR, odds ratio

In the IPMN group, 34 patients exhibited a WF or HRS and were handled as at-risk patients. Table 4 summarizes the odds of an IPMN at-risk patient having a cystic mural nodule. In total, 14 patients had a cystic mural nodule. We found that an N-shape resulted in a higher likelihood of a patient having a cystic mural nodule when compared with a descending shape (OR 5.9, *P* = 0.048). We observed no statistically significant associations for other shapes. Furthermore, no shape carried a statistically significant OR considering dilated MPD (12 cases) or an abrupt change in the MPD caliber with distal pancreatic atrophy (7 cases), or a cyst ≥ 3 cm (16 cases) as the outcomes (data not shown).

**Table 4:** Odds ratio of being an IPMN at-risk patient with a cystic mural nodule compared with the shape of the main pancreatic duct

Shape	Mural nodule				OR (95% CI)	*P* value
	Yes (n = 14)	No (n = 94)	%	Size, mm mean (range)		
Descending	5	32	13.5	14 (6–26)	1	
Vertical	1	9	10.0	13 (13)	0.93 (0.09–5.55)	0.944
Sigmoid	3	30	9.1	12 (10–13)	0.68 (0.15–2.78)	0.591
Loop type	1	3	25.0	8 (8)	2.53 (0.22–19.50)	0.411
Reverse-Z type	1	7	12.5	5 (5)	1.18 (0.11–7.31)	0.870
N-shape	3	3	50.0	7 (5–8)	5.91 (1.02–36.00)	**0.048**
Pancreas divisum	0	10	0		0.28 (0.002–2.84)	0.332

Results based on the Firth’s penalized logistic regression. Descending shape is used as a reference shape for odds ratios

CI, confidence interval; IPMN, intraductal papillary mucinous neoplasm; OR, odds ratio

In the IPMN group, 58 patients had multiple cysts that extended to three-quarters of the pancreas or across the entire pancreas. Additional file 2 presents the likelihood of being an IPMN patient with extensive cyst disease. Patients with ansa pancreatica (14 cases) were more likely (OR 12.8, *P* = 0.001) to belong to the subgroup with multiple cysts extending to three-quarters of the pancreas or across the entire pancreas compared with those who had no ansa pancreatica or no duct of Santorini. Additional file 3 presents the locations of the cysts in IPMN population and the type of the IPMN. Of the whole IPMN population 13 patients had solitary cysts which were mainly located in the body of the pancreas (9 cases). 36 patients had multiple cysts that extended to less than three-quarters of the pancreas. They were mostly located in the head and body (11 cases), and in the body and tail (9 cases), and in the uncinate process and tail (6 cases). One patient did not have cysts.

Two IPMN patients had a complex abnormal pancreaticobiliary junction connection, where a small additional connecting duct was observed between the MPD and the common biliary duct, both of which occurred in female patients. One patient had a reverse-Z type C3 subtype configuration and the other had a sigmoid configuration. Both patients had multiple IPMN cysts that extended across the entire pancreas. However, they presented with no WF or HRS.

None of the patients with an MMPD had a history of pancreatitis. Only one IPMN patient had pancreatitis in their patient history, a case that presented with a descending course in the MPD.

## Discussion

The development of IPMN disease and the reasons for why some patients develop more worrisome forms of disease remain unknown. To our knowledge, this is the first study to focus on the association between MMPD or ansa pancreatica with IPMN disease and its severity.

This study demonstrates that IPMN patients more often experience an MMPD compared to patients with a healthy pancreas (18.4% vs. 3.0%). Patients with an MMPD were more likely to present with IPMN than patients without an MMPD (OR 7.3). The ORs for loop configuration (12.4) and the reverse-Z type (OR 7.8) were also statistically significantly compared with a descending shape. Yet, the CI was rather wide for the loop configuration because the number of patients was low (only 4 cases). This warrants further study among a larger patient population. The presence of the duct of Santorini or ansa pancreatica also significantly associated with being an IPMN patient.

The N-shape did not associate with the risk of being an IPMN patient, but did carry another risk among IPMN patients. We observed an association between an N-shape and the IPMN risk in patients with cystic mural nodules. Overall three of six patients with an N-shape configuration had a cystic mural nodule, and all three patients had ansa pancreatica. In addition, five of six patients with an N-shape had ansa pancreatica, one with a duct of Santorini. In the control group, two patients had an N-shape, one with ansa pancreatica and the other with a duct of Santorini. Almost all IPMN patients with ansa pancreatica had multiple cysts extending to three-quarters or across the entire pancreas. Furthermore, 14 of 15 patients with ansa pancreatica had a wide extent of cysts. We suspect that ansa pancreatica may contribute to the extent of the cysts and the formation of cystic mural nodules. Since this is the first study to examine this specific topic, further studies are warranted. In study by Ikegawa et al. multiple cyst-existing regions correlated with the incidence of pancreatic ductal adenocarcinoma (PDAC) concomitant with IPMN, and they suspected that multifocal cysts may serve as a high-risk factor for concomitant PDAC [26].

We also compared the possible association between IPMN disease and the connection of the duct of Santorini or ansa pancreatica to the small bowel. We found that the duct of Santorini ends at the small bowel more often in IPMN patients, which associates with IPMN.

Pancreas divisum was not statistically associated with IPMN disease [27]. Interestingly, santoriniceles were only observed among IPMN patients (6 cases). In addition, one patient without pancreas divisum exhibited santorinicele, a phenomenon also reported in previous studies [22, 23].

IPMN patients had a wider MPD than the control group (3.2 mm vs 2.0 mm), a statistically significant finding. This finding may indicate that IPMN disease associates with the entire duct system. Additionally, the duct of Santorini and ansa pancreatica were visually wider in IPMN patients and, therefore, easier for a radiologist to identify.

This study carries some limitations. First, identifying control patients proved difficult. Altogether, 109 patients were excluded from the control group because of existing cysts in the pancreas. The prevalence of pancreatic cysts in the general population remains high, at 45–49%, and their number and size increase with age [28, 29]. We observed this in our study, whereby elderly patients appeared to experience pancreatic illness more often. As a consequence, our control patients were 20 years younger than the IPMN patient group. But, pancreatic anatomical variations, such as pancreas divisum or an existing duct of Santorini, do not change during one’s lifetime. Thus, these results are comparable despite the difference in patient age between groups. In the control population, three patients aged 42, 56, and 94 years old, respectively, had an MMPD, suggesting that MMPDs also exist in the younger population and may be congenital. Second, because the duct of Santorini and ansa pancreatica were wider in IPMN patients, they were also easier to follow in diagnostic images. This might result in a situation whereby the possible ending of the duct into the small bowel was not visible in some control group patients in MRCP images, warranting further studies with secretin for better duct visualization. Finally, given the retrospective design of this study, we included patients in an IPMN surveillance program for whom a histopathological confirmation was unavailable [30].

This is the first study to focus on the association between MMPD or ansa pancreatica with IPMN disease. The finding that MMPD occurs more frequently in IPMN patients may indicate that targeting IPMN follow-up in patients with an MMPD is helpful [31], although this warrants further investigation. An N-shape configuration may be associated with more severe IPMN disease, although our population was small. Thus, additional studies among larger patient population including follow-up are needed.

## Conclusions

In conclusion, IPMN patients more often exhibit an MMPD than patients with a healthy pancreas. Ansa pancreatica in IPMN patients associated with multiple cysts extending to three-quarters to across the entire pancreas. An N-shape configuration in IPMN patients positively associated with cystic mural nodules and could serve as an indication of risk among IPMN patients requiring more precise follow-up.

## Supplementary Information

**Additional file 1**. Association between the duct of Santorini or ansa pancreatica ending in the duodenum or not, and IPMN disease.

**Additional file 2**. Odds ratio of being an IPMN patient with widespread cyst disease compared with the presence of the duct of Santorini or ansa pancreatica.

**Additional file 3**. Location of the cysts in IPMN patients.

## Data Availability

The datasets generated and/or analyzed during the current study are not publicly available due to the IRB approval and privacy of patients but are available from the corresponding author on reasonable request.

## Abbreviations

BD-IPMN: Branch-duct intraductal papillary mucinous neoplasm
HRS: High-risk stigmata
IPMN: Intraductal papillary mucinous neoplasm
MMPD: Meandering main pancreatic duct
MPD: Main pancreatic duct
MRI: Magnetic resonance imaging
MRCP: Magnetic resonance cholangiopancreatography
MX-IPMN: Mixed-type IPMN
WF: Worrisome features
