# Supplementary material for: Anatomical pancreatic variants in intraductal papillary mucinous neoplasm patients: a cross-sectional study
Source: BMC Gastroenterol. 2022 Aug 21;22:394. doi: 10.1186/s12876-022-02465-w (PMC9394057; doi:10.1186/s12876-022-02465-w)
Supplement: Supplementary file 1 — Additional file 1. Association between the duct of Santorini or ansa pancreatica ending in the duodenum or not, and IPMN disease. [file 12876_2022_2465_MOESM1_ESM.docx]

Additional file 1. Association between the duct of Santorini or ansa pancreatica ending in the duodenum or not, and IPMN disease

|  |  | IPMN group  (n = 98) | Control group  (n = 100) | *P*-value | OR (95% CI) |
| --- | --- | --- | --- | --- | --- |
| Duct of Santorini |  | 55 | 30 |  |  |
|  | ends in the duodenum | 39 | 9 | **<0.001** | 5.69  (2.15–15.06) |
|  | does not end | 16 | 21 |  |  |
| Ansa pancreatica |  | 15 | 10 |  |  |
|  | ends in the duodenum | 8 | 2 | 0.108 | 4.57  (0.72–29.13) |
|  | does not end | 7 | 8 |  |  |

Logistic regression results are shown as odds ratio (OR, outcome is IPMN, control as the reference group) with 95% confidence intervals (CIs) and *P-*value. Does not end in the duodenum group is the reference. Patients with pancreas divisum excluded.

Abbreviations: CI, confidence interval; IPMN, intraductal papillary mucinous neoplasm; OR, odds ratio.
