# Supplementary material for: Anatomical pancreatic variants in intraductal papillary mucinous neoplasm patients: a cross-sectional study
Source: BMC Gastroenterol. 2022 Aug 21;22:394. doi: 10.1186/s12876-022-02465-w (PMC9394057; doi:10.1186/s12876-022-02465-w)
Supplement: Supplementary file 2 — Additional file 2. Odds ratio of being an IPMN patient with widespread cyst disease compared with the presence of the duct of Santorini or ansa pancreatica. [file 12876_2022_2465_MOESM2_ESM.docx]

**Additional file 2.** Odds ratio of being an IPMN patient with widespread cyst disease compared with the presence of the duct of Santorini or ansa pancreatica

| **Factor** | **Shape** | **OR (95% CI)** | ***P*-value** |
| --- | --- | --- | --- |
| Location  ≥75% of the pancreas |  |  |  |
|  | Ansa pancreatica | 12.76 (2.58–127.69) | **0.001** |
|  | Duct of Santorini | 1.27 (0.51–3.18) | 0.599 |
|  | Both absent |  | 1 |

Based on Firth’s penalized logistic regression analysis. Pancreas divisum patients excluded.

Abbreviations: CI, confidence interval; IPMN, intraductal papillary mucinous neoplasm; OR, odds ratio.
