# Supplementary material for: Anatomical pancreatic variants in intraductal papillary mucinous neoplasm patients: a cross-sectional study
Source: BMC Gastroenterol. 2022 Aug 21;22:394. doi: 10.1186/s12876-022-02465-w (PMC9394057; doi:10.1186/s12876-022-02465-w)
Supplement: Supplementary file 3 — Additional file 3. Location of the cysts in IPMN patients. [file 12876_2022_2465_MOESM3_ESM.docx]

**Additional file 3** Location of the cysts in IPMN patients

| Cyst location | Solitary cyst  (n = 13) | Multiple cysts extended < 75% of the pancreas  (n = 36) | Multiple cysts extended ≥75% of the pancreas  (n = 58) |
| --- | --- | --- | --- |
| Uncinate process | 1 | 2 | - |
| Head | 1 | 0 | - |
| Body | 9 | 2 | - |
| Tail | 2 | 2¹ | - |
| Uncinate process and head | - | 6 | - |
| Uncinate process and body | - | 1 | - |
| Uncinate process and tail | - | 3 | - |
| Head and body |  | 11¹ |  |
| Body and tail | - | 9 | - |
| Uncinate process, head, and body | - | - | 4² |
| Uncinate process, head, and tail | - | - | 1 |
| Uncinate process, body, and tail | - | - | 5 |
| Head, body and tail | - | - | 8¹ |
| Entire pancreas | - | - | 40⁶ |

96 patients had branch-duct IPMN, and 11 patients had mixed-type IPMN. Mixed type IPMN patients are reported in superscript as a number out of the patient proportion.

¹ one patient with mixed-type IPMN

² two patients with mixed-type IPMN

⁶ six patients with mixed-type IPMN
